# Evaluating Link Lifetime Prediction to Support Computational Offloading Decision in VANETs

**DOI:** 10.3390/s22166038

**Published:** 2022-08-12

**Authors:** Paulo Rocha, Alisson Souza, Gilvan Maia, César Mattos, Francisco Airton Silva, Paulo Rego, Tuan Anh Nguyen, Jae-Woo Lee

**Affiliations:** 1 Department of Computer Science, Federal University of Ceará, Fortaleza 60020-181, Brazil; 2 Quixadá Campus, Federal University of Ceará, Quixadá 63902-580, Brazil; 3 Virtual UFC Institute, Federal University of Ceará, Fortaleza 60020-181, Brazil; 4 Laboratory of Applied Research to Distributed Systems (PASID), Universidade Federal do Piauí (UFPI), Picos 64607-670, Brazil; 5 Konkuk Aerospace Design-Airworthiness Institute (KADA), Konkuk University, Seoul 05029, Korea; 6 Department of Computer Science and Engineering, College of Engineering, Konkuk University, Seoul 05029, Korea; 7 Department of Aerospace Engineering, Konkuk University, Seoul 05029, Korea

**Keywords:** vehicle networks (VANETs), computational offloading, machine learning, features, V2V communication, IEEE 802.11p, SUMO, NS-3

## Abstract

In urban mobility, Vehicular Ad Hoc Networks (VANETs) provide a variety of intelligent applications. By enhancing automobile traffic management, these technologies enable advancements in safety and help decrease the frequency of accidents. The transportation system can now follow the development and growth of cities without sacrificing the quality and organisation of its services thanks to safety apps that include collision alerts, real-time traffic information, and safe driving applications, among others. Applications can occasionally demand a lot of computing power, making their processing impractical for cars with limited onboard processing capacity. Offloading of computation is encouraged by such a restriction. However, because vehicle mobility operations are dynamic, communication times (also known as link lifetimes) between nodes are frequently short. VANET applications and processes are impacted by such communication delays (e.g., the offloading decision when using the Computational Offloading technique). Making an accurate prediction of the link lifespan between vehicles is therefore challenging. The effectiveness of the communication time estimation is currently constrained by the link lifespan prediction methods used in the computational offloading process. This work investigates five machine learning (ML) algorithms to predict the link lifetime between nodes in VANETs in different scenarios. We propose the procedures required to carry out the link lifetime prediction method using existing ML techniques. The tactic creates datasets with the features the models need to learn and be trained. The SVR and XGBoost algorithms that were selected as part of the assessment process were trained. To make the prediction using the trained models, we modified the lifespan prediction function from an offloading approach. To determine the viability of applying link lifespan predictions from the models trained in the road and urban scenarios, we conducted a performance study. The findings indicate that compared to the conventional prediction strategy described in the literature, the suggested link lifetime prediction via regression approaches decreases prediction error rates. An offloading method from the literature is extended by the selected SVR. The task loss and recovery rates might be significantly reduced using the SVR. XGBoost outperformed its ML competitors in task recovery or drop rate by 70% to 80% in an assessed hypothesis compared to an offloading choice technique in the literature. With greater offloading rates from an application on the VANET, this effort is intended to give better efficiency in estimating this data using machine learning in various vehicular settings.

## 1. Introduction

According to [1], the number of vehicles was calculated at 1.2 billion globally in 2014 and predicted to reach 2.0 billion in 2035. Annually, vehicles threaten the lives of about 1.3 million people around the world. It is predicted that in 2030, road accidents will be the fifth highest cause of death in the world [2]. The majority of accidents happen in urban environments and are caused by improper traffic coordination [3]. As a result, there is a need to design systems that improve traffic efficiency and safety while also offering consumers a variety of services and traffic-related information. This area has been called ITS (Intelligent Transportation Systems) [4]. A key reason for implementing ITS is the increase in the number of cars capable of connecting to massive vehicular networks and sharing data [5]. Vehicle Ad Hoc Networks (VANETs) are crucial to the deployment of ITS because they provide a variety of functions, including the supply of real-time traffic information. For example, such data may be utilised to optimise traffic flow and reduce traffic congestion [6]. Ad hoc networks, wireless LANs, mobile technologies, and sensor networks are all integrated into VANETs to enable improved intelligent communications between cars, roadside sensors, and infrastructure. This makes VANETs a versatile and complex technology [7]. Different communication channels supported by VANET include Vehicle to Vehicle (V2V), Vehicle to Infrastructure (V2I), and Hybrid channels [8]. New sophisticated applications have evolved as a result of the VANETs industry’s ongoing growth, including real-time surveillance, augmented reality, streaming video in cars, autonomous driving, and more [9]. Such applications pose a challenge to a vehicle’s constrained resources since some of them need processing power and are latency-sensitive [10].

Utilizing computational offloading techniques, which shift complete workloads to other devices to make better use of the resources available, is one approach to increase the performance of applications in VANETs. The offloading procedure in VANETs uses idle resources from neighbouring cars to conduct tasks or programmes on those devices [11]. Due to frequent route failures brought on by the high mobility of vehicles, delay and cost in computational offloading are seldom managed adequately [12]. Data packet transfers between the origin and destination vehicles frequently encounter interruptions due to the communication route’s limited lifespan, necessitating re-transmissions [13]. Offloading is strongly dependent on techniques that estimate the link lifetime because of issues with communication interruption; this is important in a computational offloading choice process in VANET [14].

Recent research views the ability to estimate the link lifespan between two vehicles as an enabler for enhancing VANET communication quality [15]. The link lifespan is not accurately predicted by the available solutions. The link lifespan prediction method is now mostly carried out by using computations and various equations, which has three primary issues or limitations. (i) Low lifetime inference accuracy can result in high error rates, or a larger difference between the predicted lifetime value and the actual value. (ii) The prediction calculus theorems only take into account a small number of features, making it challenging to incorporate new features into the prediction solution. The term “feature” in this study refers to the contextual traffic data that is gathered and used as labeled input data or variables in the prediction process. Utilizing few qualities contrasts with the development of vehicle networks, which, because of their numerous sensors and shared information, enable a more notable emergence of new features [12,16]. (iii) A small selection of analysed scenarios are either in the experiments or in the theories put out in the literature. In the data collecting stage, the majority of studies concentrates on just one kind of scenario. As time goes on, the unique traits and quirks of each vehicle’s communication environment are eventually neglected, distorting the prediction process and yielding predictions of values that are inconsistent with each context.

The lifespan prediction procedure is generally limited by machine learning (ML) techniques. Unrelated to offloading, it is noteworthy that the authors in [17] employed an ML method called Adaboost to predict the link lifespan. Other studies employ mathematical equation-based systems with a maximum of three elements that take into account the position, speed, acceleration, or direction of the vehicle. Few studies that concentrate on estimating link lifetimes make use of that data in computational offloading; examples are [14,18,19,20]; nevertheless, the settings under consideration are straightforward. Finally, apart from [21], which incorporates an urban environment into its prediction scheme, various prediction environments are not addressed, with related work concentrating primarily on the highway environment.

By changing its link lifespan prediction approach, our study attempts to expand the GCF (Greedy CPU Free) computational offloading mechanism. We suggest using ML via regression techniques in the lifetime prediction process. To train and assess learning models of various ML techniques in the literature, we want to create datasets from simulated vehicular scenarios. To accomplish the link lifetime prediction regarding linked works, we employ a larger collection of features. We conduct several performance analyses of ML algorithms using various scenario schemes, selecting the ML approach that produces the best results. Then, using the selected ML technique, we train a prediction model that we include into the computational offloading algorithm. Finally, we contrast our novel offloading strategy with previous offloading algorithms that have been proposed in the literature, including GCF, HVC (Hybrid Vehicular Cloud), and Random Choice.

To sum up, the following are the main contributions of this work:

- We propose employing eight features in the prediction process, which is a larger number than is often employed by link lifetime prediction efforts in the literature. We propose using the pseudo-angle as a feature and resource to determine the direction two cars are traveling. To determine the best effective prediction method, we examine the regression-based ML algorithms currently in use;
- Creating urban and highway scenarios utilising various data-generation techniques to anticipate the link lifetime. The public can access developed datasets and scenarios https://github.com/PauloHGR/data-sets_prediction_LLT); accessed on 1 August 2022
- We improve an offloading decision technique that was first suggested in a previous work [19] by replacing its prediction function with a more effective ML-based prediction method. The extended offloading methodology, also known as GCFML (Greedy for CPU Free with Machine Learning), is put to the test in comparison to other methods presented in the literature.

The remainder of this work is organized as follows. Section 2 addresses related work. Section 3 presents the proposal and demonstrates the tools and methodology used. In Section 4, we discuss the results of the experiments. In Section 5, we carry out a new performance evaluation, but with a new method of dataset generation and training of ML models, to improve the offloading process in the urban environment. Finally, in Section 6, we present the main conclusions and suggest directions for future work.

## 2. Related Work

Machine learning techniques are used in different ways to solve problems related to VANET. Several works emphasize using ML techniques in the decision-making optimization process in computational offloading algorithms in task offloading. The authors in [18] group vehicles to form clusters to increase the reliability of the link between nodes for efficient offloading of tasks. Next, the authors formulate an optimization problem to minimize energy consumption and task offloading latency. In [22], the authors consider collaborative computational offloading in a dynamic edge-cloud network and formulate an optimization problem in the offloading decision process. They use approaches based on deep reinforcement learning to solve the task optimization problem, thus reducing energy consumption and communication delay. Some works use machine learning approaches to predict mobility in Vanet, as in [23], which proposes a routing model based on a hybrid metaheuristic algorithm combined with ensemble learning. The authors calculate model execution using various machine learning techniques, including SVM, Naive Bayes, ANN, and decision tree. In [24], the authors implement a network traffic prediction model considering the parameters that can lead to road traffic. The proposed model integrates a random forest and network traffic prediction algorithm to simultaneously predict the network traffic flow based on road and network traffic.

As seen, the use of machine learning is quite common in the development of vehicular networks, with applications in numerous fields. However, using learning techniques in the link lifetime part is still challenging, with few works in this field. The authors do not seek to extend their applications or processes for link lifetime prediction.

We analyzed that machine learning approaches in vehicular networks are used in some fields, but there is still a shortage of work in the process of lifetime prediction. With this, we seek to raise the current state of prediction work concerning characteristics, processes, and use cases, among others. We compare some main works in the literature which implement link lifetime prediction processes with our work, highlighting characteristics that differentiate them. Table 1 summarizes the main points of analysis.

The high mobility of the vehicular scenario requires that specific features, such as speed, position, and angles, among others, be observed and considered. In [21], the authors propose a new MPBRP (Mobility Prediction Based Routing Protocol) that uses positions and angles to predict the best path.

In [25], the authors address a method that predicts the lifetime of the link between two moving vehicles based on their relative speed. Subsequently, this lifetime prediction algorithm is implemented with the Ad Hoc On-Demand Distance Vector (AODV) protocol, creating a new protocol called AODV with Lifetime Prediction (AODV-LP).

In [26], the authors propose two protocols, one to predict the most stable route and the other to predict the delivery time of packets before sending the data. A link lifetime prediction scheme was developed to guarantee the protocols’ efficiency. The authors consider the acceleration and deceleration of vehicle speed in direct communication between the two vehicles. In addition to acceleration, the speed and positioning of vehicles are features used when calculating the link lifetime. The authors consider a road scenario in the simulations, not performing tests in other scenarios, such as urban ones.

de Souza et al. [19] proposes a scheme to improve the offloading performance of computationally intensive applications while dealing with the mobility challenges of vehicular environments. The results show that the proposed scheme reduces the total download time by up to 54.1% and increases the download success rate by 71.8% compared to other schemes. However, the work does not use an efficient link lifetime prediction algorithm. Our work proposes using machine learning algorithms to more realistically predict link lifetime and thus increase offloading success rates.

In [17], a link lifetime prediction method is proposed in VANET environments using the machine learning algorithm Adaboost. A benchmark between Adaboost and other machine learning algorithms was performed, but the authors do not include link lifetime prediction solutions based on mathematical formulations in the benchmark. The simulations considered a highway environment, with urban scenarios not being used. Based on the evaluated regression metrics, our results in the highway scenario showed lower error rates than the highway scenario implemented by the authors.

In [14], a task offloading scheme is proposed in vehicular cellular communication for anything. Vehicles are grouped within the cluster, sending tasks to vehicles within the same cluster via V2V communication or with an edge server. To perform the lifetime prediction of the offloading algorithm proposed by the authors, they use a prediction function based on position, speed, and angle features. The authors consider only the highway scenario regarding the environment adopted in the experiments. In [18], the authors extend the scheme to support offloading on VEC (Vehicular Edge Computing) servers and improve the matching algorithm to optimize offloading decisions.

In [27], the authors use link lifetime prediction to identify nearby vehicles or nodes to perform a hop to a destination vehicle since location-based network protocols in VANETs employ the hop strategy to acquire the route to a node of destination. The authors propose an algorithm to calculate the lifetime of the nearby vehicle before making the jump. The authors model the proposal in a highway environment with dense traffic, considering the speed of both vehicles, the distance, and the maximum transmission range as prediction features.

In [20], the authors propose an offloading method in V2V communication by implementing an SDN (Software Defined Network) within an MEC (Mobile Edge Computing) architecture. The vehicles send their context data to the MEC, and the SDN controller calculates information regarding the ideal route between client and server vehicles. To calculate this route, the authors propose a method to calculate the link lifetime in the SDN controller. This method is calculated based on two features: relative speed and distance.

In the offloading process, the studies presented employ restricted lifespan prediction techniques. Only one study used ML-based lifespan prediction approaches; however, the study does not apply this prediction process to offloading in vehicle networks. In a decision-making approach to task offloading in vehicular communication, we apply machine learning approaches to the prediction link lifespan. The paper proposes eight aspects to address the challenge of predicting link lifespan in VANETs. Some proposed characteristics, such as pseudo-angle, angle, and SLS, have not been considered in previous publications. Another significant distinction is that we evaluate prediction efficiency using an urban scenario, which is rarely used in earlier publications due to its complexity.

## 3. Proposed Method

**Figure 1:**
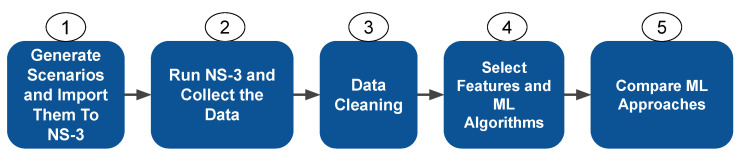
Workflow of the proposed methodology.

This section describes our methods for predicting link lifetime using machine learning. Figure 1 illustrates all the steps performed in this work. The procedure begins with the implementation of simulations using various VANET situations to collect the essential features for the learning process. Following this, we gather and calculate a set of features from vehicle communication and store the values in datasets. Each scenario creates a dataset that must be cleaned before it can be used for training. The completed datasets are then subjected to machine learning algorithms to predict the value of the link lifespan. Then, we compare the ML methods to the anticipated values using various metrics.

### 3.1. Scenario Generation

Identifying the factors that impact a feature that one wishes to assess is a crucial step in the learning process. To gather these features, we create two vehicular network scenarios: an urban and a highway scenario. Both of these situations are often employed in vehicular network applications and are also the most typical traffic scenarios; therefore, simulating these scenarios is crucial when researching methods or strategies that increase traffic mobility [28,29,30].

**Figure 2:**
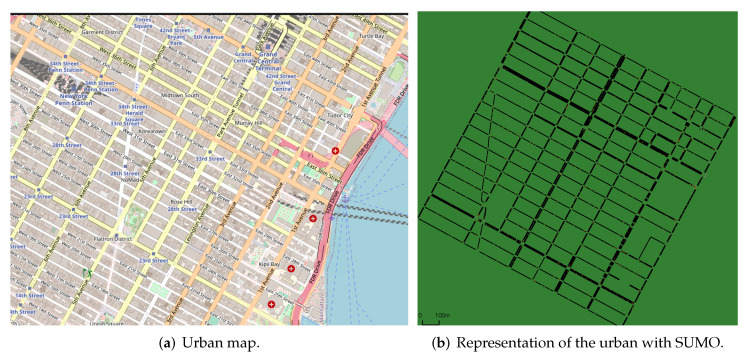
Urban scenario.

**Figure 3:**
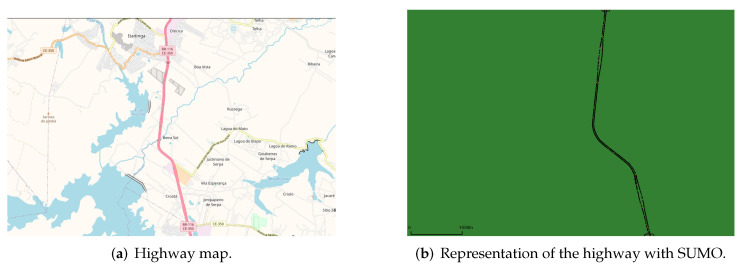
Highway scenario.

Maps of cities or portions of cities are utilised for the urban scenario, and numerous of these scenarios are employed in various simulation kinds. In many different kinds of employment, maps of cities such as Berlin, Dublin, Luxembourg, and New York are commonplace. Due to the vastness of the scenario and the high resource requirements of such situations, we selected a 2 km section of downtown Manhattan, New York, USA for this study (Figure 2a) [31,32]. As these are surroundings with little topological or route modifications, there is a greater degree of diversity in the highway scenario’s simulated locations. We chose to utilise a sample local highway with a 5 km section of the BR-116 in the city of Fortaleza, Brazil, because there are not any maps in the literature that are often used (Figure 3a). We use SUMO (https://www.eclipse.org/sumo/, accessed 1 August 2022,) and NS-3 (https://www.nsnam.org/ accessed 1 August 2022,) to run the simulated scenarios and perform the data collection step. Figure 2b and Figure 3b illustrate the SUMO representations for the considered scenarios.

### 3.2. Data Collection and Features Definition

The settings needed to set up and instantiate the scenarios in NS-3 are displayed in Table 2. Each scenario had a different amount of cars because of the variations in their topologies. Due to the significant dispersion of automobiles in the urban situation, we needed to deploy more vehicles.

Here, our aim is to provide several datasets for every scenario, which depicts various traffic situations and behaviours. Link lifetime value calculations require contextual data from the traffic environment, which the NS-3 makes possible. The first piece of information pertains to the object’s location in the plane, which is represented by the vector of coordinates $\left(P_{x},P_{y}\right)$ in this work. The other piece of information relates to the velocity vector, which in this work will be denoted by the nomenclature $\left(V_{x},V_{y}\right)$ and indicates the direction in which the object is moving.

**Figure 4:**
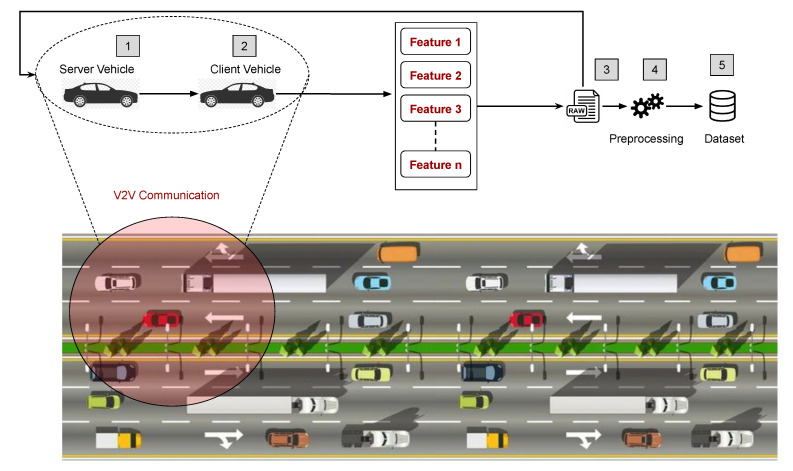
Proposed scheme for the data collection step.

The data-collecting environment is shown in Figure 4. A V2V (vehicle-to-vehicle) communication network includes the vehicles. Each car in this network requests and begins contact with other vehicles using a broadcast-based protocol. Once this contact is established, the vehicles continue to transmit and receive packets until the point at which their connectivity breaks down. The transmitting automobile *i* (client) delivers network packets with its speed and location information during this communication period at regular intervals. The receiving automobile *j* (server), upon receiving the packets, determines speed and location information in addition to other elements that will be covered later. Finally, a dataset is created by storing this data in an external file.

We give three values—0.5, 1, and 2 s—as the packet transmission interval. We split the cars into three categories and assign a transmission time to each group. By setting the simulation up with these transmission periods, significant interference and potential overloads are avoided.

According to Table 1, the research previously discussed concentrates on minor variables to prediction link lifespan. Eight characteristics are used in the prediction method in this work. They reflect elements we may gather in a real traffic situation and are traits that have been combined in many works of literature. Using the Pythagorean Theorem [33], the $D_{ij}$ distance between the two moving objects from the NS-3 is determined as follows:

(1) $$D_{ij}=\sqrt{{\left(P_{xi}-P_{xj}\right)}^{2}+{\left(P_{yi}-P_{yj}\right)}^{2}}.$$

From the velocity vector, it is possible to calculate the angles $\Theta_{i}$ and $\Theta_{j}$ related to the transmitting and receiving vehicles [34,35]:

(2) $$\Theta =\arctan\left(\frac{V_{y}}{V_{x}}\right)\frac{180}{\pi }.$$

**Figure 5:**
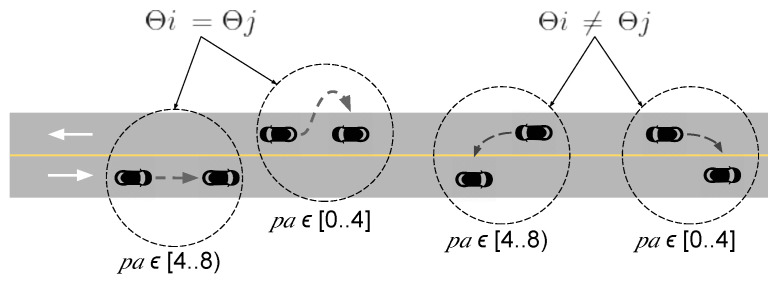
Pseudo-angle analysis for the highway scenario.

We also consider the pseudo-angle between the two vehicles as a feature, used when it is necessary to compare different angles, but without large performance costs [36,37]. We use the pseudo-angle to determine the position of the vehicle *i* regarding the position of the vehicle *j*. Figure 5 illustrates how the pseudo-angle behaves in the highway scenario. The pseudo-angle gives a number within the range [0,8), which indicates the position of *j* in the plane. We conclude that the range [0,4] indicates that *j* is behind *i*, while the range [4,8) indicates that *j* is ahead of *i*.

First, we calculate the scalar speed value extracted from the velocity vector data with the formula (3) [33]. We also consider the sum of the squared speeds *i* and *j* as another feature. The objective is to give more weight to the higher speeds or the biggest speed differences between the cars since in the simulation environment the speeds are usually similar. The new feature is calculated as follows:

(3) $$Speed=\sqrt{V_{x}^{2}+V_{y}^{2}}.$$

(4) $$S_{v}=Speed_{i}^{2}+Speed_{j}^{2}.$$

To relate speed and distance, we compute the Spatial Locality Similarity (SLS), which uses the distance and speed difference between vehicles to indicate the link length. The SLS was considered an important feature in [17]. When calculating the SLS, one must define the maximum speed and transmission range. We have set these, respectively, as $S_{\max}=17$ m/s and r=250 m. The feature is then calculated as follows:

(5) $$SLS_{ij}={\left(1+\frac{D_{ij}^{2}}{r^{2}}+{\left(Speed_{i}-\frac{Speed_{j}}{2}S_{\max}\right)}^{2}\right)}^{-1/2}.$$

We also used two other features considered in [26,38], which are the relative speed and relative acceleration of the client’s car regarding the neighboring car. These are called RS and RA and are given by:

(6) $$\begin{array}{cc} RS_{ij} & ={\left(V_{xi}-V_{xj}\right)}^{2}+{\left(V_{yi}-V_{yj}\right)}^{2}, \end{array}$$

(7) $$\begin{array}{cc} RA_{ij} & =2[\left(P_{xi}-P_{xj}\right)\left(V_{xi}-V_{xj}\right)+\left(P_{yi}-P_{yj}\right)\left(V_{yi}-V_{yj}\right)]. \end{array}$$

The communication time Tc between the vehicles *i* and *j* will be the goal variable. We will attempt to predict the value of the target or dependent variable using machine learning methods. During the simulation, we count the data that car *j* stores from each packet that car *i* delivers to determine the Tc of the two cars. Each car has a unique identification number, IdCar, which is kept in storage with the other features. The simulation also recorded the time *t* at which the vehicles *i* and *j* communicated with one another. We traverse the full dataset using the IdCar values for automobiles *i* and *j* until we reach the last entry for both cars. At this point, we identify the instant *t*. Algorithm 1 summarizes the aforementioned procedure.

**Algorithm 1:** Computation of the link duration.

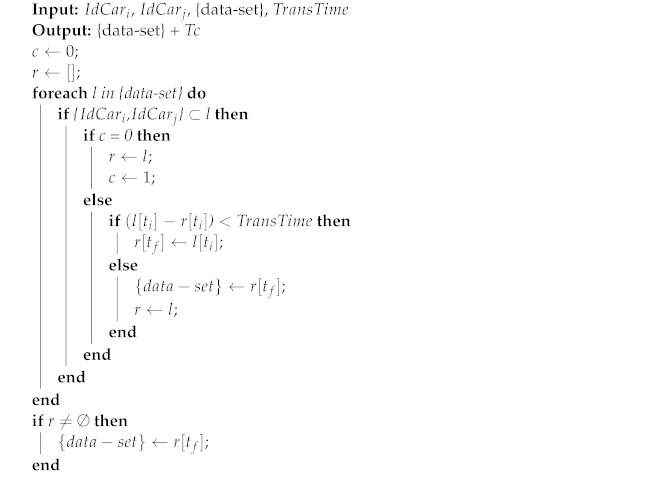



    The passing vehicle (*j*) obtains the packet from the transmitting vehicle (*i*) in the simulation at moment *t*. Two variables named $t_{i}$ and $t_{f}$ serve as redundant storage locations for the value of *t*. The method looks for the communication records of automobiles with IdCari and IdCarj in every dataset line by line (*l*). The method stores the first record in *r*, and the variable *c* obtains the value 1, signalling to subsequent interactions that the first record is in *r*. The variable TransTime refers to the transmission time of the packet. We compare the difference between the current record and the previous one with the packet transmission time. If this record difference is less than the transmission time, the cars are in communication. Then, the $t_{f}$ of the first record is updated to receive the $t_{i}$ of the current record. If the time difference of the registers is greater than the packet transmission time, there is a link drop, and the value of $t_{f}$ is updated in the dataset. When no more cars with the specified identifiers are found, the comparisons end, yielding Tc = $r[t_{f}]$.

### 3.3. Data Cleaning

Before applying the machine learning models, we pre-process the datasets to eliminate noisy data and outliers. Communication losses occur between the client and neighboring vehicles during vehicle communication, re-establishing communication immediately afterward. Thus, we consider a minimum window of 1 s during idle communication between vehicles, according to Algorithm 1. This time interval prevents new samples with a short duration from being created, thus avoiding possible distortions in the training of prediction models.

During data collection, we noticed that the samples collected did not follow a normal distribution regarding the communication time between the nodes. So we perform a feature normalization process by resizing the feature values to values within a new range from 0 to 1 using the MinMaxScaler technique [39]. We also remove samples collected with distances above 250 m, the maximum range we have configured.

### 3.4. ML Algorithms Selection

We choose the machine learning algorithms after creating and cleaning the datasets. The goal is to determine the most effective learning strategy for predicting the link lifespan under various conditions. We consider regression methods to predict communication time based on the previously detailed features through the Python 3.8 programming language. We evaluate the following techniques implemented by the scikit-learn 0.23.2 (https://scikit-learn.org/stable/, accessed on 1 August 2022) library: Epsilon-SVR (Support Vector Regression), k-Nearest Neighbors, and Random Forest. We also evaluated XGBoost version 1.4.2 (https://xgboost.readthedocs.io/en/stable/, accessed on 1 August 2022), and MLP (Multi-layer Perceptron) neural network. Table 3 shows the parameters used in the configuration of each machine learning technique.

We used the grid search method to select the hyper-parameter’s combination with the lowest prediction error rate. In the MLP, we used the Keras (https://keras.io/, accessed on 1 August 2022) through the Tensorflow v2.4 library and tested some combinations of hyper-parameters provided by Keras, with the best result shown in Table 3.

### 3.5. Evaluation of ML Approaches

For the purpose of predicting the link lifespan between nodes in vehicular networks, we compare the suggested machine learning technique with the conventional algorithm. The LLT (Link Lifetime) algorithm is used in several works and applications in the VANET literature and is well described in [26,38,40].

The following metrics were used in this work to measure the efficiency of the prediction approaches *MAE* (Mean Absolute Error), *MAPE* (Mean Absolute Percentage Error), also known as *MRE* (Mean Relative Error), and *RMSE* (Root Mean Square Error). These metrics are calculated according to Equations (8)–(10). The variable Tc is the actual value of the duration time, and the Yp is the value predicted by the solutions.

(8) $$\begin{array}{cc} \mathrm{MAE} & =\frac{1}{n}\sum_{i=1}^{n}\left|Tc-Yp\right|, \end{array}$$

(9) $$\begin{array}{cc} \mathrm{MAPE} & =\frac{100}{n}\sum_{i=1}^{n}\frac{\left|Tc-Yp\right|}{Tc}, \end{array}$$

(10) $$\begin{array}{cc} \mathrm{RMSE} & =\sqrt{\frac{1}{n}\sum_{i=1}^{n}{Tc-Yp}^{2}}. \end{array}$$

We divided the datasets for each highway and urban scenario into two additional datasets, a training dataset and a test dataset. A total of 20% of the samples are in the test data set, while the other 80% are in the training dataset. Using the previously established metrics, we assess the trained models’ predictions made using the test dataset. We utilised the values from the test dataset to compute the LLT errors throughout the simulation because we had previously computed the lifespan values using the LLT technique.

Table 4 shows the obtained metrics. Based on the high error rates indicated by the measurements, the results show that the conventional technique (LLT) delivers more extreme predictions. The predictive communication times presented by machine learning methods generalise better and are more accurate.

Finally, we measure each approach’s time to predict the link lifetime, as shown in Table 4. We calculate the time it takes to read the data and produce the prediction assuming that each machine learning model is available and installed on a server. We use a script to carry out the LLT algorithm outside of the simulator. Since the NS-3 operates in virtual time, which differs from the actual hardware running time, we conduct the measurement procedure outside of the simulation environment.

The selection of a prediction technique for computational offloading in high-traffic scenarios requires consideration of such criteria. The worst-case situation for decision making would be this since the amount of predictions is dependent on the quantity of cars that react to the discovery process.

## 4. The Impact of Link Lifetime Prediction on Computational Offloading Performance

The experiments carried out to evaluate the effects of regression-based prediction algorithms used to estimate the link lifespan between nodes in VANETs are presented in this section. In Section 4.1, we approach how the different ML algorithms evaluated in the last section impact the time required to make an offloading decision. In Section 4.2, we assess how well a computational offloading solution may perform when using the proposed prediction solution.

### 4.1. Computational Offloading Decision Time

Based on the prediction time results shown in Table 4, we ran an experiment to infer the average time each ML approach requires executing the link lifetime predictions. Finding out how long an offloading strategy will delay selecting which tasks to offload is the main objective. This is important because before making a decision the offloading algorithm must consider the anticipated timings of each surrounding automobile.

We performed simulations in highway and urban environments with high traffic, using the highest values shown in Table 5. We employed the heaviest traffic to examine the effectiveness of the ML models and LLT techniques in the worst case, when there is a larger need for the prediction models, with 500 and 605 automobiles, respectively. The average number of neighbours a vehicle encounters during the simulation is first confirmed. For the highway and urban settings, the average values that a specific car discovered from nearby vehicles were 29 and 23, respectively. Then, using the prediction times of each strategy, we realise the product of this result.

We utilise an AWS EC2 virtual machine to collect data on metrics on a vehicle-like device with limited resources. The computer runs Linux Ubuntu 20.04.2 LTS and has an Intel(R) Xeon(R) CPU E5-2650 v3 @2.30GHz, 1 CPU core, and 1 GB of RAM.

**Figure 6:**
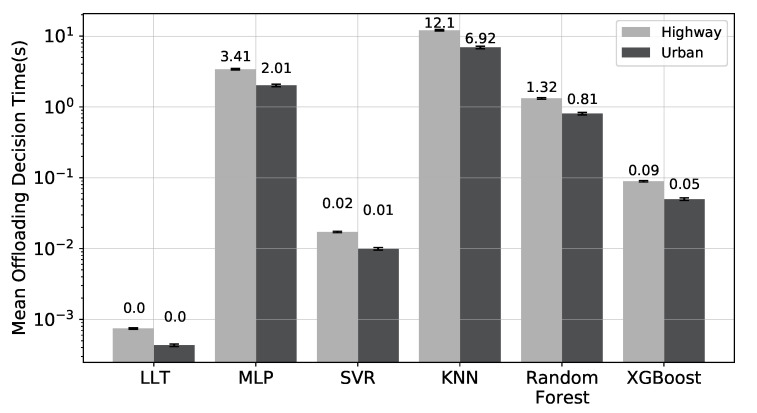
Offloading decision time for the evaluated scenarios.

Figure 6 illustrates how an offloading strategy utilising the SVR model yields a decision time that is less than that of the other ML strategies. Since LLT is a straightforward arithmetic method, its prediction time is almost nil. Since the overall decision times for the KNN, random forest, and MLP techniques are enormous, an offloading algorithm would have to wait a very long time until it had all anticipated times before making a choice.

These findings demonstrate that some ML techniques have significant overhead in situations with large traffic. The amount of time it takes to make the choice defines overhead. The decision-making process of the offloading algorithm is impacted by excessive overhead since it must wait a lengthy period. Waiting too long can lead to link breakdowns, losing touch with nearby cars, and receiving inaccurate information in a dynamic vehicular network environment. Due to the overhead they can create, even if the MLP, KNN, and random forest approaches give predictions with minimal errors, it is not practical to utilise them for offloading (at least when considering a vehicle with the computing capabilities described in the last section).

### 4.2. Computational Offloading Overall Performance

We also use actual VANETs applications to confirm the effectiveness of our ML-based link lifetime prediction. According to Figure 6, the computational offloading experiments that follow solely take into account the SVR learning model due to its strong predictive performance (low error rates) and reduced overhead in the offloading decision process in high-traffic situations.

For comparison, we choose three computational offloading algorithms: a random choice algorithm, the HVC (Hybrid Vehicular edge Cloud), and the GCF, according to [19]. These offloading algorithms use the LLT algorithm as a function to calculate the lifetime of the communication between vehicles. According to the results of the experiments presented in [19], the GCF presented the best success rates in the offloading decision process. Thus, we choose it and replace its link lifetime calculation function with the machine-learning-based approach. The resulting solution is called GCFML (GCF with Machine Learning).

We run a computational offloading simulation in the highway and urban scenarios using the NS-3 simulator. The scenarios considered in our tests were the same as the previous data collection step. In our experiments, we use two factors: the number of vehicles and task size. A vehicle is chosen at random to be the task transmitter. We use the same number of vehicles of [19,41] in the highway and urban environments, as presented in Table 5.

The objective of placing larger workloads is to test the offloading algorithms in more critical situations, which require greater efficiency in predicting the link lifetime. We use a task size value greater than in [19]. The factors with their levels for each scenario are also shown in Table 5.

On a computer with the following settings, we executed the tests: A Linux Ubuntu 18.04.2 LTS operating system is running on an Intel(R) Xeon(R) processor E5-2650 v3 CPU @2.30GHz with 8 CPU cores and 16 GB RAM.

We initially use a dataset from each scenario to train the models (highway and urban). When a client car locates a nearby vehicle, it runs a query to determine how long their relationship will last. The characteristics are gathered and sent to the model, which then provides the estimated time.

A transmitting vehicle or client delivers four tasks to nearby vehicles that can handle them under the preset offloading situation [19]. The results of tasks are returned to the client by nearby cars. Then, we assess three metrics: the success rate of offloading, the rate of retrieval, and the pace of local execution. Successful completion of the four tasks indicates that the unloading was successful. There was a gap in the link during the processing phase if any nearby vehicle did not provide the result, necessitating the need to carry out a recovery. As a result, a duplicate of that task is processed locally by the client vehicle. All tasks are completed locally in the client car when there are not enough capable vehicles to unload.

**Figure 7:**
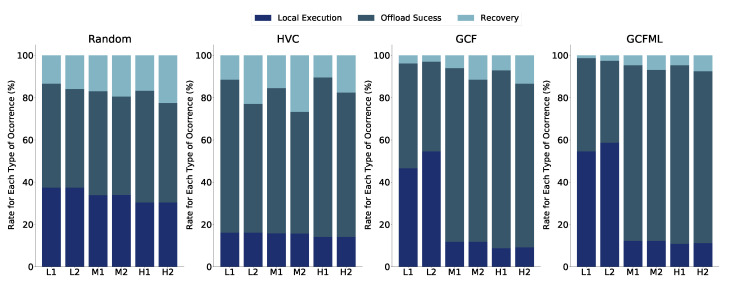
Offloading results for the highway scenario.

**Figure 8:**
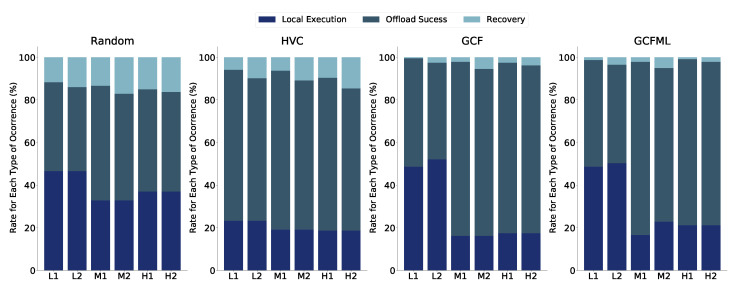
Offloading results in the urban scenario.

The results for the highway (Figure 7) and urban (Figure 8) scenarios indicate that the use of the SVR model has the potential to replace the traditional prediction approach. In comparison to its competitors, GCFML performed better in the highway scenario in terms of offloading success rates and recovery rates in the medium and high-density situations.

In scenarios with fewer tasks and heavy traffic in an urban context, our method reduced recovery rates. When deciding to execute locally rather than transmitting a task that will be lost and then recovered, GCFML makes superior offloading options. GCFML has a greater rate of local execution as a result. Another factor is the more unpredictable and dynamic metropolitan environment, which influences predictions of shorter communication times. There are more local executions because the offloading algorithm has fewer alternatives for submitting tasks with shorter link lifetime predictions.

The overall findings show that the SVR model lowers the recovery rate during the computational offloading phase in vehicular networks. Since there are fewer re-transmissions and error-correction procedures, a lower recovery rate offers applications and users a higher quality of service. The highway scenario is the most difficult prediction, with the largest recoveries at all tested levels, as shown in Figure 7. In the highway scenario, our GCFML method produced the greatest outcomes, with lower recovery rates and higher success rates. The offloading outcomes utilising the strategy based on a mathematical method are good in the urban setting despite its complicated geometry. These findings mitigate the effects of GCFML in particular urban contexts.

## 5. Improving Computational Offloading in an Urban Scenario

**Figure 9:**
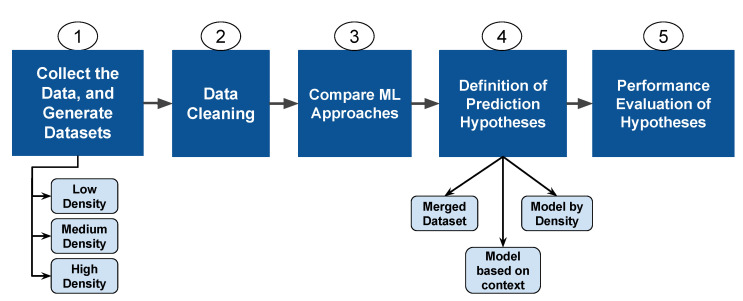
Workflow of the link lifetime prediction improvement steps.

To train our models based on traffic volumes in an urban environment, we examined novel hypotheses for the development of datasets. We created three datasets based on the low, medium, and high densities utilised in the tests in Section 4. The results in Section 4.2 demonstrated that the urban setting exhibited only modest gains in LLT efficiency. To select the method for training with the new datasets, we conducted a fresh comparison of the ML algorithms. Figure 9 illustrates the procedure used to increase lifespan prediction accuracy in an urban environment.

In the strategy used in this new link lifetime prediction procedure, we take into account a novel method of constructing datasets for ML model training. We employ new scenarios, taking into account their traffic density and dividing the datasets according to density, as opposed to only taking into consideration the scenario that is shown in Section 3.1. For each density, we utilised the number of vehicles listed in Table 5. Then, using the random selections from the runs where there were recoveries from earlier studies, we replicated each situation. These seeds allowed us to locate the precise vehicle selected at random in earlier studies. To provide more realistic datasets and maybe enhance the link lifetime prediction process in an urban setting, we generated the datasets based on the most complex scenarios. Once the datasets were prepared, they underwent a data cleaning procedure to remove noisy and distorted data. In Section 3.3, we go over the data cleaning and pre-processing stage. Based on the results of Table 4 and Figure 6, we chose the ML SVR, and XGBoost algorithms for the evaluation process and the Adaboost algorithm and its respective features based on the work of [17]. We measured *MAE*, *RMSE*, *MAPE*, the prediction time, and the size of the trained model with the results shown in Table 6.

The best results were obtained by XGBoost, as seen by the results; thus we decided to incorporate it with the GCF offloading technique. This strategy will also be known as GCFML. As demonstrated in Table 5, we experimented with this new strategy using the same variables and levels. We will monitor the average offloading rate each run, or how long it takes to accomplish the offloading, in addition to the metrics that have already been measured.

Last but not least, we tested these three assumptions for potential enhancements in offloading effectiveness in an urban environment. The first theory employed a model, which we will refer to as GCFML Merged, that was trained using just one dataset across all situations. By combining the three datasets from various traffic types, we created this unified dataset. The second assumption we put out involved employing unique models for each density. Therefore, we employ the model trained with a dataset created based on that density, which we refer to as GCFML Density, for each scenario performed with that density. The last hypothesis that was put to the test was employing the GCFML Zone model, which was based on the density of the client vehicle’s present position.

In this last hypothesis, we want to analyse it from the perspective of the client vehicle rather than employing a distinct ML model for each scenario based on the density of the entire scenario. The goal is to utilise a certain model dependent on the volume of traffic near the vehicle. The client car transmits discovery packets through broadcasting to determine how many nearby vehicles it has.

**Figure 10:**
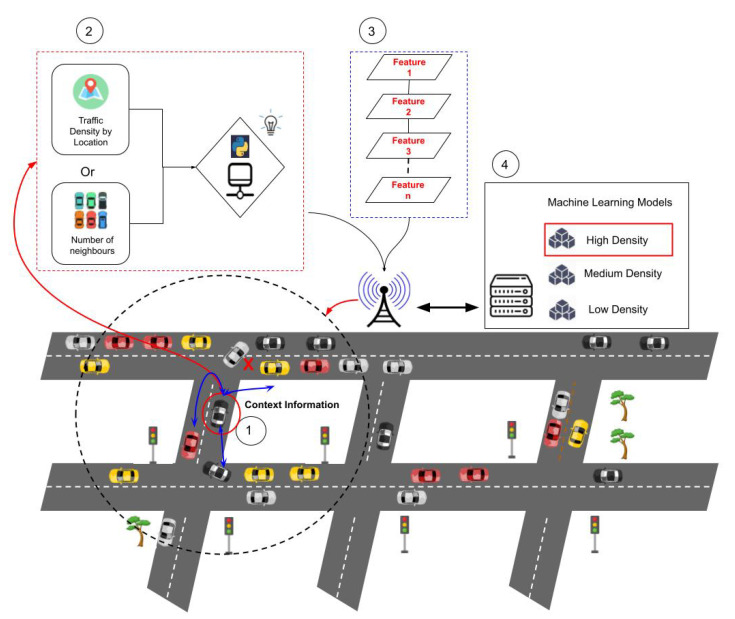
Flow of the prediction process with different context approaches.

The second and third hypotheses’ execution flows are shown in Figure 10, where the vehicle also collects the context data required to determine which trained ML model to employ for prediction in addition to the characteristics utilised in link lifetime prediction. There are three models available, each of which was trained using datasets gathered in three situations with various traffic volumes.

Step 1 begins with the customer completing a context knowledge procedure. The second hypothesis of the experiments pertains to the selection of a particular ML model based on each scenario, which in this case would be a geographic region. In this situation, the client gathers the Geo-location data. The client broadcasts packets to find the number of adjacent cars that are appropriate for unloading if the third hypothesis is correct. We utilise the work values of [42] to determine the traffic density the automobile is in based on the reactions of nearby cars. Finally, using the context data gathered for the relevant hypotheses, an application selects the ML model for the prediction (step 2). The client contacts nearby automobiles at the same time as the model decision process is taking place to gather the data needed for the ML model to make the prediction (step 3). In step 4, the client vehicle delivers the data from the selected model and the gathered characteristics to a storage server for the trained models, which might be a cloud server, an edge server, or even the automobile itself. The server selects the ML model, completes the prediction process, and provides the client with the projected communication time value (step 5).

**Figure 11:**
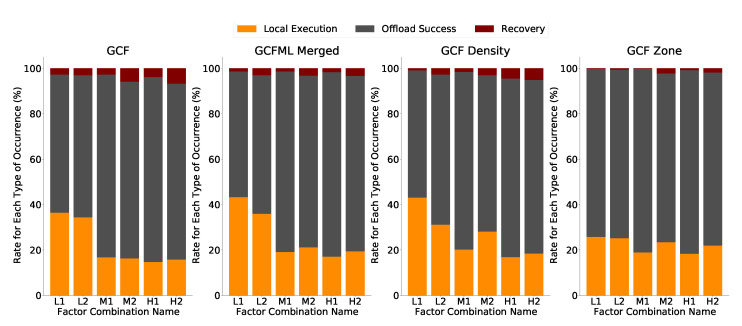
Comparison of offloading completion rates across all approaches.

The findings in Figure 11 demonstrate a decline in the rate of recoveries for each hypothesis considered. The rate of local executions fell around the GCF when we used the model for the third hypothesis based on local density or the customer vehicle location zone. The most notable decline in recoveries in relation to the GCF method was in the GCFML Zone. In the H2 scenario, the difference has caused the absolute number of recoveries to decline by roughly 5%. The reduction in the number of retrievals from the GCFML Zone pertaining to the GCF in other situations with the task size at 558 KB is higher than 80% in the scenarios that were examined.

The number of tasks completed in the client vehicle, or the local execution rate, is another measurement that is examined. In the most low-traffic circumstances (L1 and L2), the GCFML Zone decreases local executions by up to 30% with respect to the GCF. The vehicle may occasionally fail to communicate tasks to capable surrounding vehicles, causing local execution rates to be false negative. In the GCF L1 and L2 situations, there are more local executions, which might mean that there are more false negatives. The GCFML Zone made the decision to carry out the recovery process in the other scenarios because the GCFML Zone in those scenarios somewhat increased the number of local performances, which was associated to a fall in the recovery rate. The GCFML Zone made the decision to carry out the task locally in these scenarios rather than sending it to a vehicle that would not finish the processing in the other scenarios that were examined. This increase in the number of local performances was linked to a slight decrease in the recovery rate.

Related to GCFML Merged, there is a decrease of 50% in the number of recoveries concerning the recovery rate of the GCF in all scenarios except L2. Despite the better effectiveness in identifying cars that could not process the tasks, the GCFML Merged approach showed increases in the number of local executions. This increase was not offset by the decrease in the number of recoveries, indicating that this solution had a higher incidence of false negatives in the sparse scenario (L1 and L2). In the other scenarios (M1, M2, H1, H2), the number of local task executions was not high concerning the GCF. Therefore, for scenarios with higher vehicle traffic and consequent availability of resources, GCFML Merged is acceptable.

Finally, in the second hypothesis (GCFML Density), the number of local runs decreases to 70% concerning the GCF in the first scenario (L1). In the other scenarios, there were smaller reductions of 43% and 48% in the M1 and M2, respectively, against the GCF recovery values. However, there was an increase in all scenarios in the number of local executions, negatively impacting the offloading rate of tasks performed in this approach. Therefore, the GCFML Density proved to be the least indicated solution with the other solution hypotheses in improving the offloading process in an urban scenario.

We conclude that this new approach to building and training ML models, with different datasets grouped by density, proved much more effective in offloading efficiency. Depending on the scenario, choosing an ML model that generalizes the density of the entire scenario can present some challenges, as shown in the results of the second hypothesis. Therefore, solutions such as the third hypothesis evaluated (Zone GCFML), which considers the awareness of the vehicle context, choosing the trained model according to the availability of neighboring cars, can improve the prediction process.

**Figure 12:**
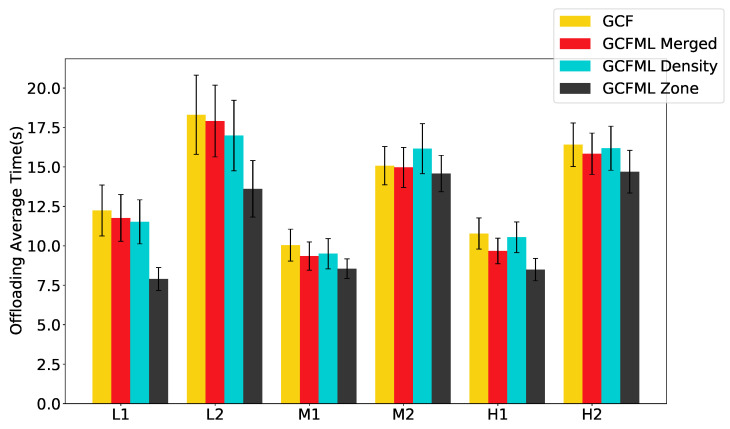
Time offloading of the approaches.

In these new experiments, we analyzed the impact of these approaches on the average offloading time, as shown in Figure 12. The improvement in the offloading success rate reflects in the offloading time since a lower number of recoveries means a lower number of copy reruns on the client vehicle. Thus, there is a reduction in offloading time with less local processing since neighboring vehicles have an excellent distribution of tasks. With this parallelism, there is naturally a lower latency.

Due to the smaller number of vehicles present in the low-density scenarios (L1 and L2), there is a longer average offloading time, which causes more local executions in the client vehicle due to the low number of neighboring vehicles. This greater granularity between local execution, recovery, and offloading rates causes more significant time variability in both offloading algorithms (confidence interval in Figure 12).

The GCFML Zone proved to be the most efficient approach in performing offloading with a shorter processing time than the GCF, especially in L1, L2, and H1 scenarios. We performed a Friedman statistical test with a confidence interval of 95% to verify the possibility of time differences in the other scenarios in which the confidence interval overlapped, with these results presented in Table 7. We concluded that in scenarios M1 and H2, the GCFML Zone had a lower average offloading time than the GCF. In the M2 scenario, there was a tie between all the approaches analyzed, as the p-test values were more significant than 0.05.

In the M1 and H1 scenarios, the first hypothesis (GCFML Merged) demonstrated a superior execution rate than the GCF, indicating that this approach is applicable to scenarios with more traffic and smaller workloads. The findings from the second hypothesis (GCFML Density) were the worst of the other hypotheses, showing that selecting prediction models based only on the traffic density scenario is ineffective for predicting link lifespan. Finally, we draw the conclusion that the GCFML Zone is the best suitable method to predict the link lifespan in urban settings based on the reported offloading time rates and the lower recovery rates.

## 6. Conclusions

This study looked at estimating the connection lifespan between nodes in VANETs using regression-based prediction approaches. This kind of technique is essential for computational offloading scenarios because it enables the transfer of jobs to stronger machines, advancing technology and enhancing the efficiency of the applications that rely on computational offloading. The experimental findings on simulated datasets show the potential of regression-based machine learning techniques such as SVR. They increase prediction effectiveness, increasing the accuracy of the values. Finding characteristics that affect connection longevity also turned out to be a valuable input to subsequent discoveries. With lower recovery rates than LLT, the SVR regression model was extended to a computational offloading approach and improved the decision-making process. Finally, in preparation for future work, we intend to test new prediction hypotheses based on training with more precise situations. Verifying if models for specific scenarios may outperform more generic models, such as those based on the highway and urban scenarios, will be the main objective.

## Figures and Tables

**Table 1:** Comparison with related work.

Work	Prediction Features	Scenario	Does It Use ML in the Prediction?	Does It Use Offloading?	Contribution
[21]	Distance, Direction, and Speed	Urban	No	No	A new protocol called MPBRP (Mobility Prediction Based Routing Protocol) seeks to predict the mobility of nodes through positions and angles to predict the best route.
[25]	Relative Speed and Position	Highway	No	No	A method predicts the TVE between two moving vehicles based on their relative speed.
[26]	Position, Speed, and Acceleration	Highway	No	No	Implementation of two protocols. One is to predict the most stable route, and another is to predict the time of packet delivery before sending the data. A prediction scheme of LLT was developed, aiming to guarantee the efficiency of protocols.
[14,18]	Position, Speed, and Angle	Highway	No	Yes	Task offloading scheme for cellular vehicle to everything (C-V2X). Performance evaluation with other offloading schemes through a road scenario simulation.
[27]	Speed, Distance, and Maximum Transmission Range	Highway	No	No	Introduction of a model to use the Residual Link Lifetime and one-hop latency in VANET.
[20]	Speed and Distance	Highway	No	Yes	A offloading method in V2V communication by implementing an SDN (Software Defined Network) within a MEC (Mobile Edge Computing) architecture, abbreviated as the SDNi-MEC server.
[19]	Position, Speed, and Acceleration	Highway	No	Yes	A scheme to improve offloading performance in multi-interface networks, through a greedy choice algorithm called GCF.
[17]	Link Lifetime, SLS, Function Similarity, Relative Mobility, Distance, and Speed	Highway	Yes	No	The authors implemented an LLT prediction method in VANET environments using the Adaboost Machine Learning algorithm. They performed a benchmark between Adaboost and other ML methods.
This work	Distance, Client Angle, Server Angle, Pseudo- angle, Speed, Relative Speed, Relative Acceleration, and SLS	Highway and Urban	Yes	Yes	We propose a prediction scheme of the Link Lifetime through the SVR Machine Learning algorithm, chosen through a performance evaluation among other ML methods. We extend the GCF algorithm with a SVR-based prediction function.

**Table 2:** NS-3 settings.

Parameter	Value
Scenario	Urban and Highway
Simulation Time	85 s
Vehicles	100 (Highway) e 200 (Urban)
Transport Protocol	UDP
Radio Propagation Model	Two-Ray Ground
Wireless Access Protocol	IEEE 802.11p
Transmission Range	250 m
Data Rate	27 Mbps

**Table 3:** ML Models’ hyper-parameters.

Algorithm	Hyper-Parameters
MLP	loss_function: *MAE*, optimizer: Adam, metrics: *MSE*, *MAE*, epochs: 1500
SVR	C: 8, epsilon: 0.05, gamma: 1.0, kernel: RBF
KNN	n_neighbors: 13, weights:distance, n_estimators: 500
Random Forest	max_depth: 100, max_features: 3, min_samples_leaf: 2, min_samples_split: 8, n_estimators: 500
XGBoost	colsample_bytree: 0.8, learning_rate: 0.1, max_depth: 8, min_child_weight: 3, n_estimators: 150, nthread: 4, subsample: 0.8

**Table 4:** Summary of the experimental results.

Algorithm	Scenario	*MAE* (s)	*MAPE* (%)	*RMSE* (s)	Prediction Time (s)
LLT	Highway	24.14	208.86	39.59	2.25× $10^{-5}$
MLP	Highway	1.89	34.55	3.13	1.03 × $10^{-1}$
SVR	Highway	1.82	35.53	3.00	5.20 × $10^{-4}$
KNN	Highway	1.99	37.29	3.12	3.66 × $10^{-1}$
Random Forest	Highway	2.01	38.03	3.15	4.00 × $10^{-2}$
XGBoost	Highway	2.07	37.04	3.24	2.70 × $10^{-3}$
LLT	Urban	25.68	190.44	40.08	2.25× $10^{-5}$
MLP	Urban	1.65	19.31	2.96	1.05 × $10^{-1}$
SVR	Urban	1.85	21.84	2.92	5.20 × $10^{-4}$
KNN	Urban	1.93	22.64	2.90	3.61 × $10^{-1}$
Random Forest	Urban	1.84	20.43	2.79	4.20 × $10^{-2}$
XGBoost	Urban	1.96	20.94	2.95	2.60 × $10^{-3}$

**Table 5:** Factors and levels considered in the experiments.

Density	#Vehicles in Urban	#Vehicles in Highway	Task Size	Name
Low	50	55	1.5 MB	L1
Low	50	55	3.0 MB	L2
Medium	275	275	1.5 MB	M1
Medium	275	275	3.0 MB	M2
High	509	605	1.5 MB	H1
High	509	605	3.0 MB	H2

**Table 6:** Summary of the new hypothesis’s experimental findings.

Algorithm	Density	*MAE* (s)	*MAPE*	*RMSE* (s)	Prediction Time (s)	Size of Model
SVR	low	3.23	0.47	4.13	0.58	53.2 KB
XGBoost	low	2.34	0.28	3.20	0.64	787. 7 KB
Adaboost	low	4.12	0.62	5.03	0.61	48.1 KB
SVR	medium	5.13	1.10	6.69	0.57	64 KB
XGBoost	medium	4.11	1.00	5.55	0.69	1.6 MB
Adaboost	medium	4.58	1.27	5.61	0.63	59.5 KB
SVR	high	4.10	0.43	5.37	0.57	22.8 KB
XGBoost	high	3.63	0.35	4.53	0.65	1 MB
Adaboost	high	4.34	0.48	5.33	0.62	70 KB

**Table 7:** Statistical Tests of the Average Offloading Time in the Scenarios.

Approaches	L1	L2	M1	M2	H1	H2
GCF × GCFML Merged	1.000	1.000	0.038	1.000	0.002	0.658
GCF × GCFML Density	0.624	1.000	0.005	0.903	1.000	1.000
GCF × GCFML Zone	0.000	0.000	0.009	0.107	0.000	0.000
GCFML Merged x GCFML Density	1.000	0.904	1.000	0.380	0.012	0.320
GCFML Merged × GCFML Zone	0.000	0.001	0.181	0.644	0.012	0.021
GCFML Density × GCFML Zone	0.000	0.012	0.278	0.408	0.000	0.001

## Data Availability

https://github.com/PauloHGR/datasets_prediction_LLT, accessed on 1 August 2022.

## Abbreviations

The following abbreviations are used in this manuscript:

MDPI	Multidisciplinary Digital Publishing Institute
ITS	Intelligent Transportation Systems
VANET	Vehicular Ad Hoc Network
V2V	Vehicle to Vehicle
VEC	Vehicular Edge Computing
MEC	Mobile Edge Computing
LLT	Link Lifetime
ML	Machine Learning
SVR	Support Vector Regression
HVC	Hybrid Vehicular Cloud
GCF	Greedy CPU Free
GCFML	Greedy CPU Free with Machine Learning
NS-3	Network Simulator 3
SUMO	Simulation of Urban Mobility
*MAE*	Mean Absolute Error
*MAPE*	Mean Absolute Percentage Error
*RMSE*	Root Mean Square Error
